# Multifunctional 3D matrixes based on flexible bioglass nanofibers for potential application in postoperative therapy of osteosarcoma

**DOI:** 10.1093/rb/rbae088

**Published:** 2024-07-24

**Authors:** Lihuan Wang, Liting Yuan, Yanbing Dong, Wenli Huang, Jichang Zhu, Xuexian Du, Chenglin Zhang, Pengbi Liu, Jinpeng Mo, Bingyan Li, Zijin Liu, Xi Yu, Hui Yu

**Affiliations:** Guangdong-Hong Kong Joint Laboratory for New Textile Materials, School of Textile Science and Engineering, Wuyi University, Jiangmen 529020, China; Guangdong Laboratory of Chemistry and Fine Chemical Industry Jieyang Center, Jieyang 515200, China; Guangdong-Hong Kong Joint Laboratory for New Textile Materials, School of Textile Science and Engineering, Wuyi University, Jiangmen 529020, China; Guangdong-Hong Kong Joint Laboratory for New Textile Materials, School of Textile Science and Engineering, Wuyi University, Jiangmen 529020, China; Guangdong-Hong Kong Joint Laboratory for New Textile Materials, School of Textile Science and Engineering, Wuyi University, Jiangmen 529020, China; Guangdong-Hong Kong Joint Laboratory for New Textile Materials, School of Textile Science and Engineering, Wuyi University, Jiangmen 529020, China; Guangdong-Hong Kong Joint Laboratory for New Textile Materials, School of Textile Science and Engineering, Wuyi University, Jiangmen 529020, China; Guangdong-Hong Kong Joint Laboratory for New Textile Materials, School of Textile Science and Engineering, Wuyi University, Jiangmen 529020, China; Guangdong-Hong Kong Joint Laboratory for New Textile Materials, School of Textile Science and Engineering, Wuyi University, Jiangmen 529020, China; Guangdong-Hong Kong Joint Laboratory for New Textile Materials, School of Textile Science and Engineering, Wuyi University, Jiangmen 529020, China; Guangdong-Hong Kong Joint Laboratory for New Textile Materials, School of Textile Science and Engineering, Wuyi University, Jiangmen 529020, China; Guangdong-Hong Kong Joint Laboratory for New Textile Materials, School of Textile Science and Engineering, Wuyi University, Jiangmen 529020, China; Guangdong-Hong Kong Joint Laboratory for New Textile Materials, School of Textile Science and Engineering, Wuyi University, Jiangmen 529020, China; Guangdong Laboratory of Chemistry and Fine Chemical Industry Jieyang Center, Jieyang 515200, China; Guangdong-Hong Kong Joint Laboratory for New Textile Materials, School of Textile Science and Engineering, Wuyi University, Jiangmen 529020, China; Guangdong Laboratory of Chemistry and Fine Chemical Industry Jieyang Center, Jieyang 515200, China

**Keywords:** bioglass nanofibers, 3D matrixes, photothermal therapy, osteosarcoma, bone defects

## Abstract

Postoperative treatment of osteosarcoma is one of the major challenging clinical issues since both elimination of residual tumors and acceleration of bone regeneration should be considered. Photothermal therapy has been widely studied due to its advantages of small side-effect, low-toxicity, high local selectivity and noninversion, and bone tissue engineering is an inevitable trend in postoperative treatment of osteosarcoma. In this study, we combined the tissue engineering and photothermal therapy together, and developed a kind of multifunctional nanofibrous 3D matrixes for postoperative treatment of osteosarcoma. The flexible bioactive glass nanofibers (BGNFs) prepared by sol–gel electrospinning and calcination acted as the basic blocks, and the genipin-crosslinked gelatin (GNP-Gel) acted as the cement to bond the BGNFs forming a stable 3D structure. The stable porous 3D scaffolds were obtained through ice crystal templating method and freeze-drying technology. The obtained GNP-Gel/BGNF 3D matrixes showed a nanofibrous structure that highly biomimetics the extracellular matrix. The excellent compression recovery performance in water of these matrixes made them suitable for minimally invasive surgery. In addition, these 3D matrixes were not only biocompatible *in vitro*, but also benefit for the formation of mineralized bone *in vivo*. Furthermore, the dark blue GNP-Gel also acted as the photothermal agent, which endowed the GNP-Gel/BGNF 3D matrixes with efficient photothermal antitumor and photothermal antibacterial performance without addition of other toxic photothermal agents. Therefore, this study provides an ingenious avenue to prepare multifunctional nanofibrous 3D matrixes with photothermal therapy for postoperative treatment of osteosarcoma.

## Graphical abstract

**Figure:**
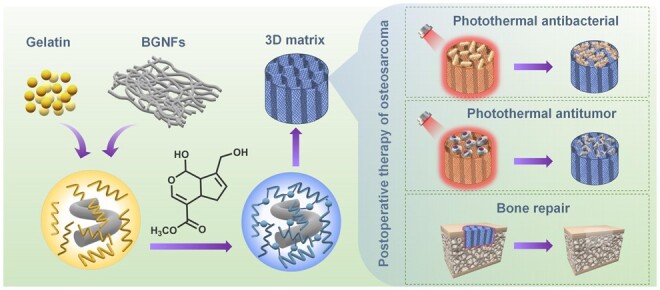

## Introduction

Osteosarcoma is a kind of notorious bone cancer and its victims are usually young people at the age of 10–25. The treatment of osteosarcoma is challenging mainly for two reasons: (I) Destructively critical bone defects are formed after the surgical resection of osteosarcoma, which are hard to self-heal. (II) The incomplete resection of tumor would exacerbate high recurrence rate, which requires postoperative chemotherapy or radiotherapy. However, the toxic drugs of chemotherapy and radiation hazards of radiotherapy damage healthy tissue/organs and inhibit the rapid bone regeneration [1, 2]. Therefore, for better postoperative therapy of osteosarcoma, both elimination of residual tumors and acceleration of bone regeneration should be considered.

To effectively inhibit tumor, photothermal therapy taking advantages of small side-effect, low-toxicity, high local selectivity and noninversion has been widely studied [3, 4]. Commonly, photothermal agents, such as gold-based materials [5], carbon-based materials [6], metal sulfides [1], etc., are prepared as nanoparticles, and then injected or transported to the tumor sites. However, photothermal therapy using nanoparticles alone is not suitable for postoperative therapy of osteosarcoma, since blood vessels in the bone defect site are absent, which limits the distribution of nanoparticles. In addition, the critical-sized bone defects after oncologic resection cannot heal spontaneously, which usually require bone substitution or osteogenic matrixes to help bone repair [7, 8]. Therefore, multifunctional bone substitutions which could inhibit residual tumors and promote bone defect repair simultaneously have been widely considered as a kind of promising materials for tumor-induced bone defect therapy. Wu *et al.* have successively combined different types of photothermal materials, including graphene oxide [9], Fe_3_S_4_ microflower [10], SrCuSi_4_O_10_ nanosheets [11], Fe_3_O_4_ microparticles [12], etc. with 3D printing technology, developing a variety of dual-function scaffolds. In addition, Maleki *et al.* incorporated Bi_2_S_3_ nanoribbons [13] and Ti_3_C_2_ nanosheets [14] into 3D printed hydrogel scaffolds, and the obtained scaffolds could effectively inhibit tumor cells and promote bone conduction. These studies have confirmed the feasibility of combining tissue engineering with photothermal therapy in the postoperative treatment of osteosarcoma.

Nanomaterials have unique physical, chemical and biological properties, including high specific surface area, excellent optical properties, good biocompatibility, etc., and these advantages make nanomaterials showing a broad application prospects in many aspects, such as drug delivery, cancer treatment, biosensing and detection, and tissue engineering [15–18]. Nanofibrous 3D matrixes have drawn extensive attention of scholars in the biomedical field since the nanofibrous structure is biomimetic to the physical microstructure of extracellular matrix (ECM), and significantly contributes to cell adhesion, proliferation, migration and differentiation [19]. In recent years, 3D porous nanofiber matrixes with stable structure were prepared through nanofiber-dispersion, freeze-drying and bonding/crosslinking, and the bulk density, morphology and mechanical properties of these matrixes were adjustable, showing great potential in the field of tissue engineering [20, 21]. In our previous works, 3D matrixes based on SiO_2_ and SiO_2_–CaO nanofibers were fabricated according to this method, and the nanofiber structure and inorganic components were proved could effectively promote osteogenesis [22–24].

**Scheme 1.**
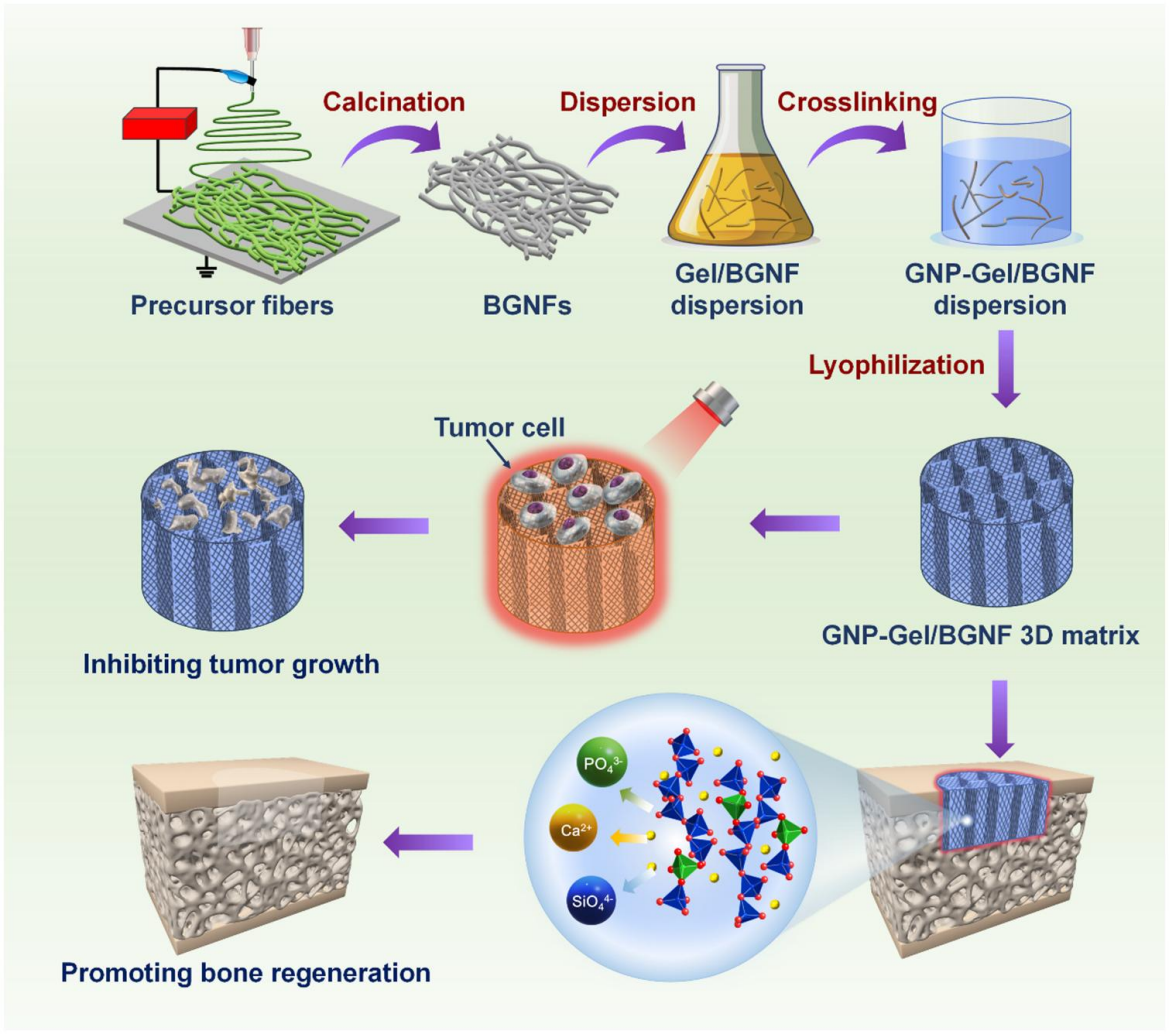
Schematic of GNP-Gel/BGNF 3D matrix fabrication and the potential applications in postoperative treatment of osteosarcoma.

Therefore, in this study, we combined the 3D nanofiber matrixes and photothermal therapy together to fabricate a kind of multifunctional 3D matrixes for postoperative treatment of osteosarcoma. Bioactive glass nanofibers (BGNFs) fabricated by sol–gel electrospinning and calcination acted as the basic blocks, and the genipin-crosslinked gelatin (GNP-Gel) acted as the cement to bond the BGNFs forming stable 3D structure. Bioactive glass, a kind of Class A bioactive materials, exhibits excellent bioactivity, osteoconductivity and osteoinductivity, has been widely employed in clinical bone repair [25]. Gelatin showing excellent biocompatibility and biodegradability, also has been extensively used for diverse pharmaceutical and medical applications [26]. Genipin, a natural cross-linker with negligible cytotoxicity, is commonly used in herbal medicine, preparation of gelatin capsules and the immobilization of enzymes [27, 28]. Exquisitely, dark blue pigment is obtained when genipin reacts with amino acids of gelatin, which can act as the photothermal agent without addition of other toxic photothermal agents [28]. Therefore, the GNP-Gel/BGNF matrixes are speculated performing excellent antitumor efficacy and osteogenic capability, and will be a kind of promising material for the therapy of bone defects after tumor resection (Scheme 1).

## Materials and methods

### Materials

Triethyl phosphate (TEP), ethyl silicate and gelatin were purchased from Maclean Biochemical Co., Ltd, calcium nitrate tetrahydrate (Ca(NO_3_)_2_·4H_2_O) and acetic acid were purchased from Guangzhou Chemical Reagent Factory, anhydrous ethanol was purchased from Guangdong Guanghua Technology Co., Ltd, genipin was acquired from Saen Chemical Technology (Shanghai) Co., Ltd, phosphate buffer saline (PBS) powder was purchased from Ubio Technology Co., Ltd, osteosarcoma (U-2OS) cells were purchased from Kefan (Guangzhou) Technology Co., Ltd, pancreatic enzyme solution was purchased from Shanghai Hongsheng Biotechnology Co., Ltd and Cell Counting Kit (CCK-8) was purchased from Beyotime Biotechnology Co., Ltd.

### Fabrication of BGNFs by sol–gel electrospinning and calcination

The precursor solution for electrospinning was prepared as follows: Firstly, the inorganic sol was obtained by addition of ethyl silicate, TEP, Ca(NO_3_)_2_·4H_2_O, ultrapure water and acetic acid in anhydrous ethanol with a stir for 2 h. Then the inorganic sol was mixed with 10 wt% PVB solution with a further stir for 6 h. The electrospinning process was operated at the following parameters: The voltage was 20 kV, the tip to collector distance was 20 cm, the infusion velocity was 1 ml/h, the ambient temperature was 20 ± 5°C and the relative humidity was 50 ± 5%. The obtained precures fiber mats were placed in a vacuum oven for 48 h, and then transferred to the Muffle furnace for calcination. The obtained BGNFs with a designed SiO_2_/CaO/P_2_O_5_ weight ratio of *x*/95–*x*/5 were referred to as *x*Si (*x *=* *50, 60, 70, 80 and 90), and the content of each reagent in the inorganic sols is listed in Table 1.

**Table 1.** The content of each reagent in the inorganic sols

	50Si	60Si	70Si	80Si	90Si
Anhydrous ethanol (g)	10	10	10	10	10
H_2_O (g)	1.5	1.5	1.5	1.5	1.5
Acetic acid (g)	0.5	0.5	0.5	0.5	0.5
TEP (g)	0.36	0.36	0.36	0.36	0.36
Ethyl silicate (g)	4.2	4.2	4.9	5.6	5.6
Ca(NO_3_)_2_·4H_2_O (g)	5.255	4.13	2.95	1.725	0.60

### Fabrication of GNP-Gel/BGNF 3D matrixes by nanofiber reconstruction

First, the BGNF mats were cut into small pieces (about 5 mm × 5 mm) and dispersed in ultrapure water by dispersion equipment to get short fiber dispersion, and then the short fiber dispersion was filtered by 100 mesh sieve, and the short fibers left on the sieve were further dried by lyophilization. Second, 1 wt% gelatin solution was prepared by adding 0.1 g of gelatin in 9.9 g of ultrapure water and heating at 40°C, and 0.6 wt% genipin solution was obtained by dissolving 0.06 g of genipin in 9.94 g of ultrapure water. Third, 1 wt% gelatin solution, 0.6 wt% genipin solution, ultrapure water and short BGNFs were mixed according to Table 2 and stirred for 4 h. Finally, the mixed dispersions were transferred into molds and frozen in −80°C refrigerator for 2 h, and then lyophilized for 48 h.

**Table 2.** The content of each matter in the dispersions for GNP-Gel/BGNF 3D matrixes fabrication

Genipin content	1%	2%	4%	8%
0.6% GNP solution (g)	0.05	0.1	0.2	0.4
1% gelatin solution (g)	3	3	3	3
Ultrapure water (g)	1.88	1.83	1.73	1.53
Short BGNFs (g)	0.07	0.07	0.07	0.07

### The mineralization experiments of BGNFs

The *in vitro* mineralization experiments of BGNFs were operated by immersing 10 mg of BGNFs in 10 ml of simulated body fluid (SBF) for 5 days, and the SBF was prepared by addition of the solutions listed in Table 3 in sequence. Before adding NaHCO_3_ solution, 30 ml of deionized water was added, and then 0.56 ml of NaHCO_3_ solution was added. Finally, the deionized water was added to constant volume line of 50 ml volumetric bottle.

**Table 3.** The content and sequence of each salt solution during SBF preparation

Sequence	Chemical compound	Concentration	Volume (ml)
1	NaCl	0.5 M/l	10
2	CaCl_2_	0.1 M/l	1.25
3	MgCl_2_	0.1 M/l	0.25
4	NaH_2_PO_4_·H_2_O	0.1 M/l	0.5
5	KCl	0.1 M/l	0.25
6	NaHCO_3_	0.75 wt%	0.56

### Photothermal property test of GNP-Gel/BGNF 3D matrixes

To test the photothermal performance of GNP-Gel/BGNF 3D matrixes, samples with a size of about 2 mm × Φ5 mm both in dry and wet (sample soaked in 200 μl water) were irradiated with an 808 nm near-infrared laser (0.9–2 W cm^−2^). The temperature was record when it was no longer rising.

### The *in vitro* biocompatibility and photothermal therapy assay of GNP-Gel/BGNF 3D matrixes

For biocompatibility and photothermal therapy assay, 1 × 10^4^ U-2OS cells were cultured on each sterilized GNP-Gel/BGNF matrix (Φ 5 mm × 2 mm) in the 96-well plate, and 200 μl culture medium was added in each well. The cells were cultured at 37°C, 5% CO_2_ and the medium was changed every two days. After two days of culture, half of the samples were irradiated by 808 nm near-infrared irradiation (NIR, 2 W cm^−2^) for 5 min. The cell viability was assayed by CCK-8 and the live/dead fluorescent staining.

### The photothermal bacteriostasis evaluation of GNP-Gel/BGNF 3D matrixes

The photothermal bacteriostasis evaluation was operated by immersing the GNP-Gel/BGNF 3D matrix (about Φ 5 mm × 2 mm) in 200 μl of bacterial (*Staphylococcus aureus* or *Bacillus coli*) suspension, and half of the samples were treated by NIR (808 nm, 2 W cm^−2^) for 5 min (NIR group), and the other half samples were not treated by NIR (No NIR group). For comparison, black groups (bacterial suspensions without GNP-Gel/BGNF 3D matrixes) with the same operations were added. Next, the samples were incubated at 37°C for 4 h, and then the bacterial suspensions of all groups were diluted 20 times, followed by spreading 50 μl of diluted bacterial suspensions on each agar dish. After 24 h of incubation at 37°C, the area of colony was analyzed by Image J.

### The *in vivo* bone regeneration evaluation

The animal experiments comply the ethics of Institutional Animal Care and Use Committee (ethical approval number is IAC23W147). The *in vivo* bone regeneration experiments were taken on Sprague Dawley (SD) rats (Zhuhai BesTest Bio-Tech Co., Ltd) at the age of 12 weeks. First, the SD rat were anesthetized by intraperitoneal injection of Sumianxin II (Dunhua City Shengda Animal Medicine Co., Ltd), and cranial defects with a diameter of 6 mm were created by trephine, followed by implanting GNP-Gel/BGNF 3D matrixes into the cranial defects. Here, we took GNP-Gel 3D matrixes as the control group. Bone regeneration after 12 weeks of surgery was characterized by a micro-CT (Bruker). 3D reconstruction was accomplished by CT-Vox (Bruker), and bone volume relative to tissue volume (BV/TV) and bone mineral density (BMD) were quantitative analyzed by CT-Scan (Bruker). The histological evaluations were taken after 12 weeks of repair. The rat calvariums were decalcified at first, and then embedded in paraffin, sliced into sections and de-paraffinized with xylene. Finally, the de-paraffinized tissue sections were stained with Masson staining.

### Characterization

The morphologies of BGNFs, minerals on BGNFs and GNP-Gel/BGNF 3D matrixes were characterized by scanning electron microscope (SEM, HITACHI). The element components of BGNFs and minerals on BGNFs were tested by energy dispersive spectrometer (EDS, HITACHI). The chemical and physical structures were analyzed by Fourier transform infrared spectrometer (FTIR, Nicolet iS50, Thermo Fisher Scientific), Raman (Renishaw, Via Qontor-EL6000) and X-ray diffraction (XRD, Rigaku, Ultima IV). The mechanical property of GNP-Gel/BGNF 3D matrixes was tested by universal testing machine (compression speed was 50 mm min^−1^).

## Results and discussion

### The flexibility, morphology and bioactivity of BGNFs

**Figure 1.**
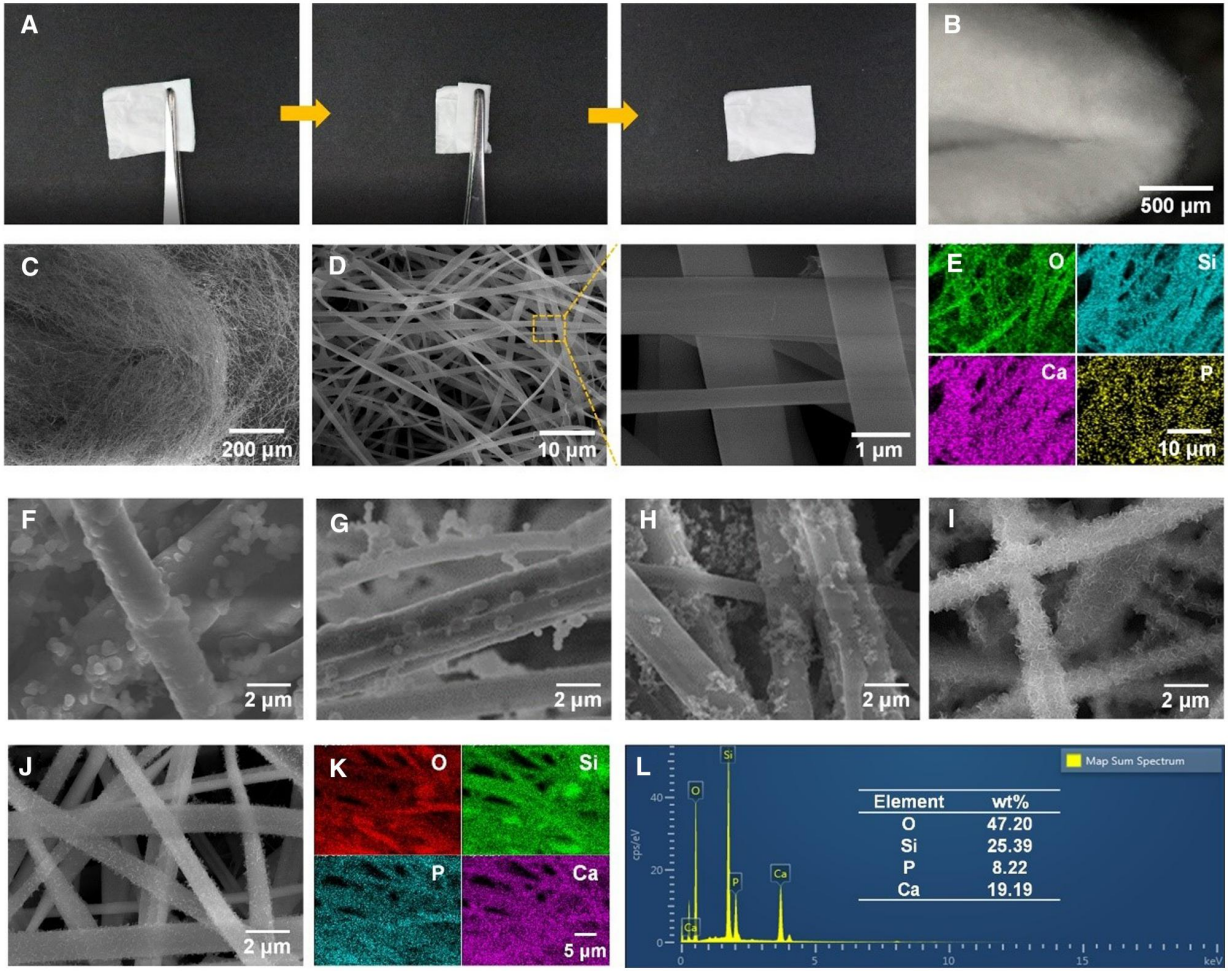
The flexibility, morphology and bioactivity of BGNFs. (**A**) The physical display, (**B**) ultra-depth-of-field microscope image, (**C, D**) SEM images and (**E**) EDS elemental mapping of 80Si BGNFs calcinated at 800°C. The SEM images of mineralized BGNFs: (**F**) 50Si, (**G**) 60Si, (**H**) 70Si, (**I**) 80Si and (**J**) 90Si. (**K**) The EDS elemental mapping and (**L**) line scan of mineralized 80Si BGNFs calcinated at 800°C.

Figure 1A and Supplementary Figure S1 show the bending test images of the BGNF membranes calcinated at 800°C. It can be clearly seen from the photographs that the BGNF membranes of 80Si and 90Si maintained good structural integrity even after bending. Here, we took the 80Si as an example to analyze the flexibility of BGNF membranes by observing the fiber state at the bending site (Figure 1B and C). It can be observed that there were few damages at the bending site, and the fibers at the bending site also maintained a relatively continuous state, indicating the fiber membrane has good flexibility. In addition, the microtopography of the BGNF membranes with various SiO_2_ contents and calcinated at different temperatures were observed by SEM. As shown in Figure 1D and Supplementary Figure S2, it was obviously seen that as the SiO_2_ content increasing, the average fiber diameter of BGNFs calcinated at 800°C decreased gradually, and the number of fiber breakage reduced. And as the calcination temperature increasing, the number of fiber breakage increased. The BGNFs with higher CaO content (50Si and 60Si) even exhibited melting phenomena when calcinated at 900 and 1000°C, making the BGNF membranes brittle. These phenomena are consistent to the macroscopic performance that BGNF membranes with higher CaO content or calcinated at higher temperature were more fragile. The EDS element mapping images (Figure 1E) showed that O, Si, Ca and P were evenly distributed in the BGNFs, which are the major components of bioactive glass.

It is reported that the Ca^2+^ and ${\text{PO}}_{4}^{3-}$ released from the bioactive glass can promote biomineralization and further facilitate bone formation [29]. Herein, we conducted mineralization experiments by soaking 10 mg of BGNFs in 10 ml of prepared SBF for 5 days, and then biomineralization activity was evaluated by observing the mineralized layer on the surface of BGNFs. As shown in Figure 1F−J, the minerals on the BGNFs calcinated at 800°C increased firstly and then decreased as the SiO_2_ content increasing, and the content of minerals on the 80Si BGNFs was the highest. The element mapping of minerals on 80Si BGNFs (Figure 1K) showed Ca and P were uniformly dispersed, and the element content data (Figure 1L) showed that the weight ratios of Ca and P were 19.19% and 8.22%, respectively, which were much higher than the theoretical weight ratios of Ca and P (10.7% and 1.1%) in 80Si BGNFs before mineralization, demonstrating the calcium phosphate minerals were formed on the BGNFs surface. Furthermore, minerals on 80Si BGNFs calcinated at different temperature were also characterized (Supplementary Figure S3), and the minerals on BGNFs calcinated at 800°C exhibited a higher uniformity and larger content than that on BGNFs calcinated at 900 and 1000°C.

### Physicochemical structure of BGNFs

**Figure 2.**
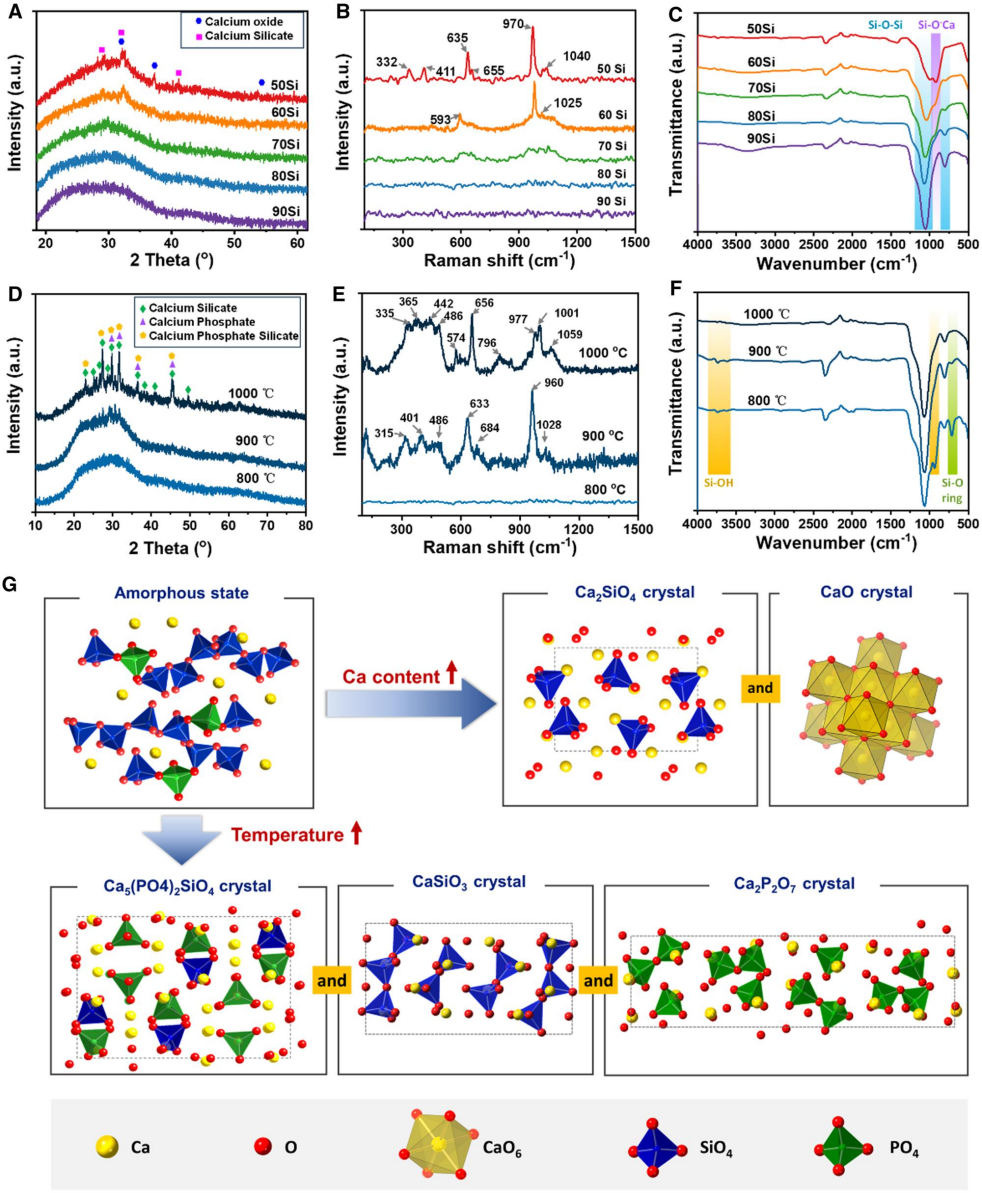
Physicochemical structure of BGNFs. (**A**) XRD, (**B**) Raman and (**C**) FTIR of BGNFs calcinated at 800°C. (**D**) XRD, (**E**) Raman and (**F**) FTIR of 80Si BGNFs calcinated at different temperatures. (**G**) The schematic of phase transition as the element content and calcination temperature changing.

To gain a deeper understanding of the fundamental reasons for the flexibility and mineralization behaviors of the BGNFs, XRD, Raman and FTIR characterizations were operated. Figure 2A–C show the XRD, Raman and FTIR spectra of BGNFs with various SiO_2_ contents calcinated at 800°C, respectively. From the XRD spectra (Figure 2A), a bread peak was observed in the range of 15–50° for all samples, indicating the phases of all samples calcinated at 800°C were mainly amorphous. As the content of SiO_2_ decreasing, shark peaks at about 29.3°, 32.2°, 37.3°, 41.2° and 53.9° appeared and enhanced gradually, which correspond to the formation of calcium silicate (Ca_2_SiO_4_) and calcium oxide (CaO) crystals. The crystallinity of 50Si, 60Si, 70Si, 80Si and 90Si obtained through XRD full spectrum peak fitting analysis were 2.78%±0.01%, 2.61±0.67%, 0.93%±0.13%, 0.46%±0.15% and 0.00%±0.00%, respectively (Supplementary Figures S4–S8).

In Figure 2B, few shark peaks were observed from the Raman spectra of 70Si, 80Si and 90Si, indicating the phase of these samples were amorphous, which is consistent with the XRD data. Moreover, for 50Si and 60Si, Raman band at ∼332 cm^−1^ assigned to CaO stretching vibrations, band at ∼410 cm^−1^ ascribed to symmetrical stretching of ${\text{PO}}_{4}^{\;3-}$, band at ∼593 cm^−1^ ascribed to the asymmetric bending of ${\text{PO}}_{4}^{\;3-}$ [30], band at ∼633 cm^−1^ assigned to the stretching vibration of Si_2_$O_{6}^{\;4-}$ [31, 32], band at ∼970 cm^−1^ attributed to the symmetrical stretching of ${\text{PO}}_{4}^{\;3-}$ and stretching vibration of Si_2_$O_{6}^{\;4-}$ [31–34], and bands at 1025 and 1040 cm^−1^ correspond to asymmetrical stretching vibrations of ${\text{PO}}_{4}^{3-}$ [33] were observed, also indicating the calcium silicate and calcium oxide crystals formed.

FTIR data of BGNFs calcinated at 800°C showed that with the decrease of SiO_2_ content, the peak at 797 cm^−1^ weakened and disappeared progressively [29]. Similarly, the intensity of peak in the range of 1000–1070 cm^−1^ also decreased gradually. In contrast, the peak around 920 cm^−1^ appeared and enhanced eventually [29, 35, 36]. It is recognized that the peak in the vicinity of 797 cm^−1^ is attributed to Si–O–Si bonds, while the peaks at 1000–1070 cm^−1^ are attributed to Si–O–Si and ${\text{PO}}_{4}^{\;3-}$ vibrations, and the peak at 920 cm^−1^ is associated with Si–O–Ca vibrations. These data indicate that as the CaO content increases, the ratio of non-bridging oxygen atoms in the SiO_2_ network increased, and the degree of polymerization of SiO_2_ network decreased.

Keeping the element content constant (taking 80Si as an example), and elevating the calcination temperature, it was found from the XRD spectra in Figure 2D and Supplementary Figures S9 and S10 that the crystallinity of the BGNFs gradually improved, being 3.38%±0.42% (calcinated at 900°C), 19.59%±0.65% (calcinated at 1000°C), respectively. And the main compositions of the crystals were calcium phosphate (Ca_2_P_2_O_7_), calcium silicate (CaSiO_3_) and calcium phosphate silicate [Ca_5_(PO_4_)_2_SiO_4_] crystals, which can be concluded from the shark peaks appeared at 23.1°, 25.2°, 25.6°, 27.2°, 29.1°, 29.8°, 30.6°, 31.7°, 36.4° and 45.5°. The Raman bands of 80Si calcinated at 900°C appeared at 315, 401, 486, 574, 633, 684, 960 and 1028 cm^−1^, and the Raman bands of 80Si calcinated at 1000°C appeared at 335, 365, 442, 486, 574, 656, 796, 977, 1001 and 1059 cm^−1^ (Figure 2E), also indicating the calcium phosphate, calcium silicate and calcium phosphate silicate related crystals were formed [37–40]. Besides, with the elevation of calcination temperature, the peak at 3500–3900 cm^−1^ (Si–OH) in the FTIR spectra (Figure 2F) became evident, and novel peaks at 710 and 930 cm^−1^ relating to the Si–O–Ca vibration emerged and intensified [29, 35, 36]. On the contrary, the peak at 797 cm^−1^ (Si–O–Si chain) weakened progressively [29]. The FTIR data revealed that the SiO_2_ network undergoes a transformation that Si–O–Si chains gradually contracted to form Si–O–Si rings. During this contraction, some Si–O–Si chains were broken, resulting in the formation of Si–OH groups.

Therefore, the effects of SiO_2_ content and calcination temperature on the flexibility of BGNFs could be concluded as follows (Figure 2G): (I) for BGNFs calcinated at 800°C, as the content of CaO increases, the degree of polymerization of SiO_2_ network decreased, and the fractures and defects increased during the process of crystallization, resulting in the flexibility of BGNFs decreased [41, 42]. (II) For BGNFs with fixed element content (80Si), as the calcination temperature increases, the crystallinity increased sharply, which led to the BGNFs fragile. Meanwhile, the SiO_2_ network undergoes a transformation that part Si–O–Si chains were broken as the calcination temperature increasing, which also led to the BGNFs fragile.

The mineralization occurred when the ${\text{SiO}}_{4}^{\;4-}$, Ca^2+^ and ${\text{PO}}_{4}^{\;3-}$ ions leaching from the BGNFs combined with Ca^2+^ and ${\text{PO}}_{4}^{\;3-}$ ions in the SBF and deposited on the fiber surface. Therefore, the mineralization activity of BGNFs should be related to the Ca content and the degree of crystallinity: (I) For BGNFs calcinated at 800°C, as the content of Ca increases, the release of Ca^2+^ increased, thus the minerals on 80Si BGNFs were more than that on 90Si BGNFs. However, since the calcium silicate and calcium oxide crystals were formed in 50Si, 60Si and 70Si BGNFs, the release of Ca^2+^ from these fibers were hindered. Finally, the minerals on 50Si, 60Si and 70Si BGNFs were less than 80Si BGNFs. (II) For 80Si BGNFs calcinated at various temperature, the content of minerals decreased as the temperature elevating since the crystallinity increased. Interestingly, though the crystallinity of 80Si calcinated at 1000°C was much higher than that of 50Si, 60Si and 70Si BGNFs calcinated at 800°C, but the minerals were more. This phenomenon should be attributed to the large number of defects in 80Si BGNFs calcinated at 1000°C, which led to more Ca^2+^ leaching. Therefore, 80Si BGNFs calcinated at 800°C with the best flexibility and bioactivity were chosen as the basic blocks for GNP-Gel/BGNF 3D matrixes construction.

### Physicochemical and photothermal properties of GNP-Gel/BGNF 3D matrixes

**Figure 3.**
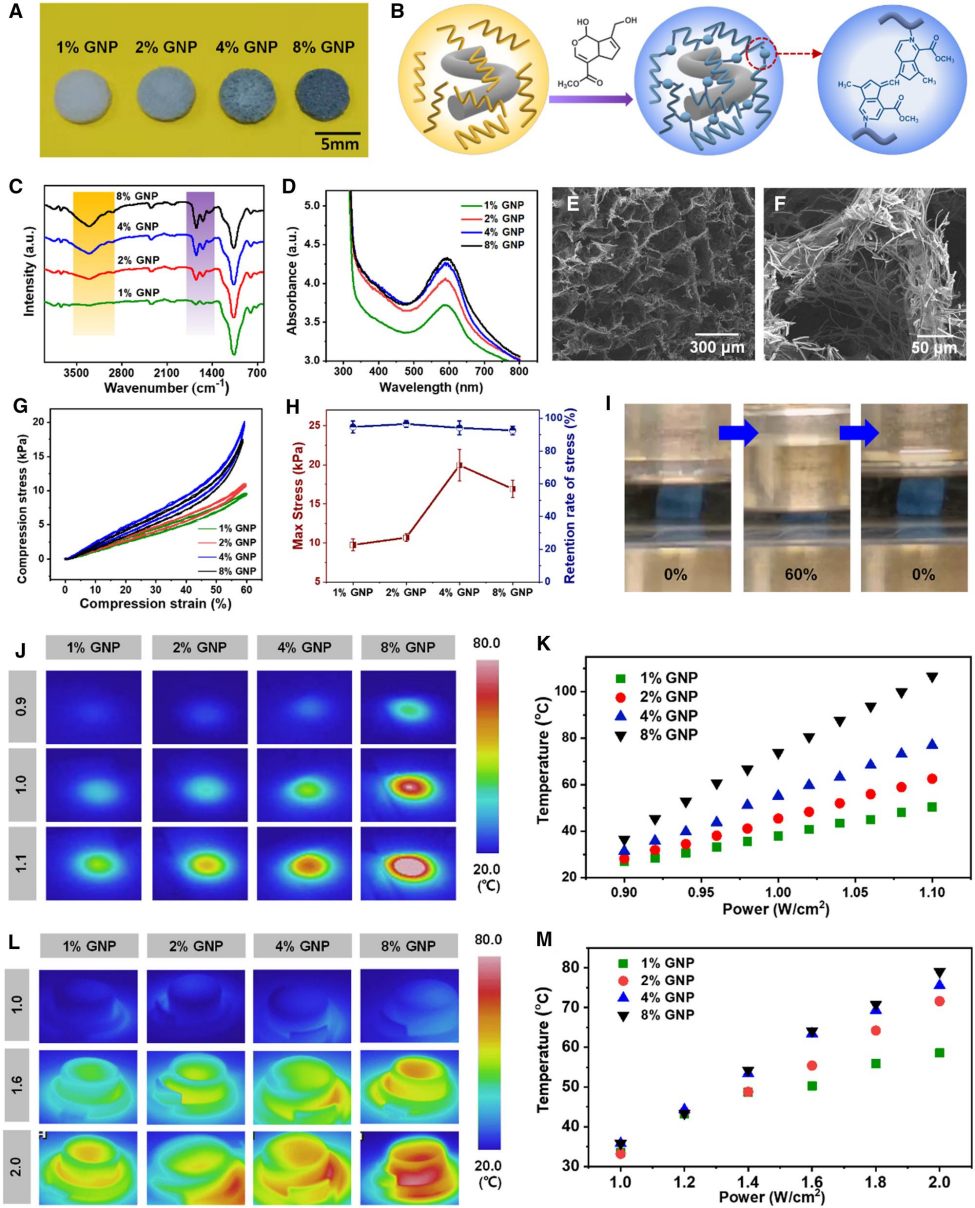
Physicochemical properties of GNP-Gel/BGNF 3D matrixes. (**A**) Digital photo of GNP-Gel/BGNF 3D matrixes. (**B**) Schematic of crosslinking reaction between genipin and gelatin. (**C**) FTIR and (**D**) UV–vis spectra of GNP-Gel/BGNF 3D matrixes. (**E, F**) The microstructure of GNP-Gel/BGNF 3D matrix with 4% genipin. (**G**) The cyclic compression stress-strain curves and (**H**) maximum stresses and stress retention rates of GNP-Gel/BGNF 3D matrixes after 10 cycles of compression. (**I**) Compression display of GNP-Gel/BGNF 3D matrix with 4% genipin in water. (**J**) The thermal images and (**K**) temperature data of GNP-Gel/BGNF 3D matrixes in dry upon NIR. (**L**) The thermal images and (**M**) temperature data of GNP-Gel/BGNF 3D matrixes in water upon NIR.

Figure 3A shows the physical images of GNP-Gel/BGNF 3D matrixes with different genipin content. The bluish color was formed after gelatin reacting with genipin. The schematic diagram of the crosslinking reaction of genipin with gelatin is shown in Figure 3B. Since the dispersion of BGNFs was basic (pH = 8.4), nucleophilic attack of genipin by the OH^−^ in the dispersion led to a ring-opening reaction, and an intermediate aldehyde group was formed, followed by a Schiff reaction between the aldehyde groups on the polymerized genipin and amino groups on the gelatin, forming a crosslinked structure [43]. We further analyzed the crosslinking structure and color by FTIR and UV–vis spectroscopy, respectively. It can be seen from Figure 3C that the peak at 3300 cm^−1^ representing the C–H bond of alkyne and the peaks at 1650, 1540, and 1441 cm^−1^ representing the aromatic hydrocarbon became stronger gradually as the content of genipin increases, and the blue color (590 nm) of the matrixes also became deeper as the content of genipin increases (Figure 3A and D), indicating more crosslinking structures were formed.

The microstructure of the GNP-Gel/BGNF 3D matrix (4% GNP) is shown in Figure 3E and F. A porous structure with a pore size of 50–300 μm was observed, and the pore walls were formed by short BGNFs and GNP-Gel, which provided a nanofibrous network structure for cell adhesion. Subsequently, we tested and analyzed the mechanical properties of the GNP-Gel/BGNF 3D matrixes. It can be seen that the compression stress–strain curves of 10 cycles were almost overlapped for all samples (Figure 3G), and the stress retention rate after 10 cycles of compression was larger than 95% (Figure 3H). The compression and resilience process of GNP-Gel/BGNF 3D matrix under water displayed in Figure 3I also showed there was little deformation. All these results demonstrated that the GNP-Gel/BGNF 3D matrixes exhibited excellent compression recovery performance in water, which made them suitable for minimally invasive surgery. When the genipin content was 4%, the compressive stress of the sample was the maximum (20 kPa). It was speculated that the crosslinking structures were not enough when the genipin concentration was lower than 4%, and overmuch when the genipin concentration was 8%. Since the compressive stress of the 3D matrix with 4% genipin is similar to that of collagenous bone tissue (>20 kPa) [44, 45], it is presumed that it can promote osteogenesis.

The obtained dark blue GNP-Gel can act as the photothermal agent [28], therefore, we tested the photothermal properties of the GNP-Gel/BGNF matrixes. Figure 3J–M shows the infrared imaging pictures and temperature data of the 3D matrixes with different genipin contents in both dry and wet states. It can be seen that the higher the content of genipin, the higher the photothermal temperature. The matrix with 8% genipin reached a highest temperature of 74°C under 1 W cm^−2^ of NIR in dry. In comparison, the matrix with 8% genipin reached a highest temperature of 78°C in 120 s under 2 W cm^−2^ of NIR in water (Figure 3M and Supplementary Figure S11). Therefore, the GNP-Gel/BGNF 3D matrixes are suit for photothermal treatment of tumor.

### Biocompatibility and photothermal inhibition of tumor and bacteria properties of GNP-Gel/BGNF 3D matrixes

**Figure 4.**
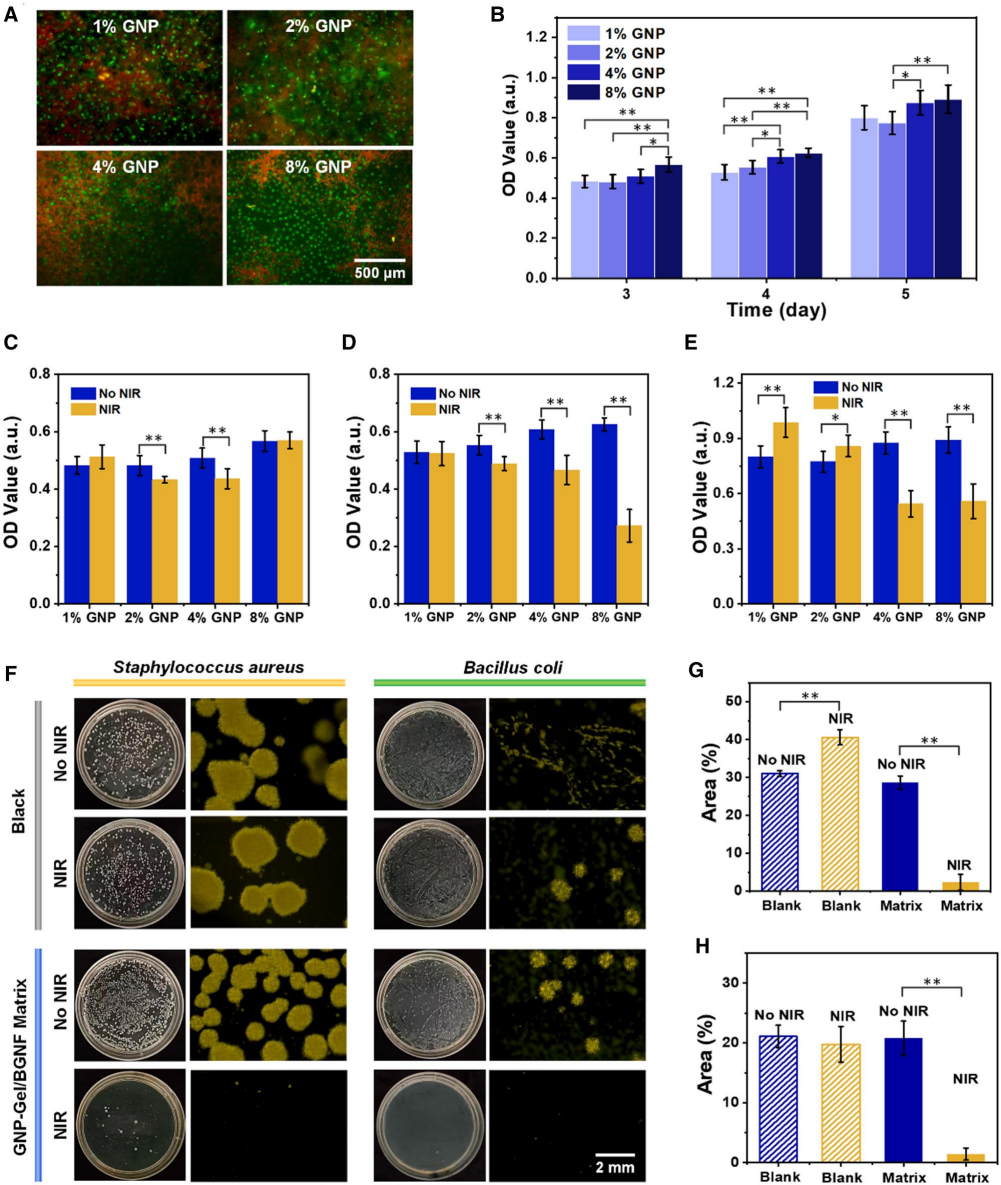
Biocompatibility and photothermal inhibition of tumor and bacteria properties of GNP-Gel/BGNF 3D matrixes. (**A**) Live/dead staining and (**B**) cell viabilities of U-2OS cells on GNP-Gel/BGNF 3D matrixes. Cell viabilities of U-2OS cells on GNP-Gel/BGNF 3D matrixes at the (**C**) 1st, (**D**) 2nd and (**E**) 3rd day after the NIR treatment. (**F**) Photographs of the bacteria colonies. Colony areas of (**G**) *Staphylococcus aureus* and (**H**) *Bacillus coli* with and without NIR treatment. Data are presented as mean ± SD. *P <* *0.05, **P <* *0.01.

The biocompatibility of GNP-Gel/BGNF 3D matrixes was characterized by the live/dead staining and CCK-8 assay. It is obvious that there were a large number of living cells (green) on all GNP-Gel/BGNF 3D matrixes with various genipin contents (Figure 4A), indicating the biocompatibility of all GNP-Gel/BGNF 3D matrixes was favorable. Quantitatively, the CCK-8 results showed the cell viability increased as the incubating time increasing, and the viability of cells on the 3D matrixes with more genipin was higher than that with less genipin (Figure 4B). These results were consistent with the reports that the cytotoxicity of genipin-crosslinked products is very low, and it is estimated to be approximately 10 000 times less cytotoxic than glutaraldehyde [27, 46]. Thus, choosing genipin as the crosslinking agent was helpful to improve the biocompatibility of the matrixes.

Based on the good photothermal performance of the matrixes, we conducted photothermal inhibition of tumor cells experiments and photothermal sterilization experiments. Figure 4C–E displays the cell viability at the 1st, 2nd and 3rd day after NIR (that was the 3rd, 4th and 5th day after seeding cells onto the matrixes). The cell viability of all the NIR groups showed no significant difference with the No NIR groups at the 1st day, but significantly lower than that of the No NIR groups with 4% and 8% genipin at the 2nd and 3rd day, and the inhibition rates of 4% and 8% GNP at the 3rd were 37.7% and 37.4%, respectively. Thus, the photothermal performance of matrixes with 4% and 8% GNP could inhibit tumor cells effectively. According to Song’s report, the *in vivo* tumor photothermal therapy was effective when the genipin-crosslinked nanoparticles were injected into to the mice body [47]. Therefore, GNP-Gel/BGNF 3D matrixes were expected and could effectively inhibit the residual osteosarcoma.

Considering both the mechanical properties and tumor inhibition performance, we took 4% GNP crosslinked matrix for the photothermal sterilization experiments. To avoid the thermal effect purely caused by NIR laser, we added black groups (no matrixes were immersed in the bacteria solutions) for comparison. As shown in Figure 4F–H, numerous *S. aureus* and *B. coli* colonies were formed on the agar medium for black groups with NIR or No NIR, thus the thermal effect purely caused by NIR laser can be ignored. In the matrix groups, the total colonies areas of both bacteria in the NIR groups were much smaller than that in the No NIR groups, and the photothermal inhibition rate of *S. aureus* and *B. coli* was about 92% and 93%, respectively, showing an excellent photothermal sterilization property.

### *In vivo* bone regeneration properties of GNP-Gel/BGNF 3D matrixes

**Figure 5.**
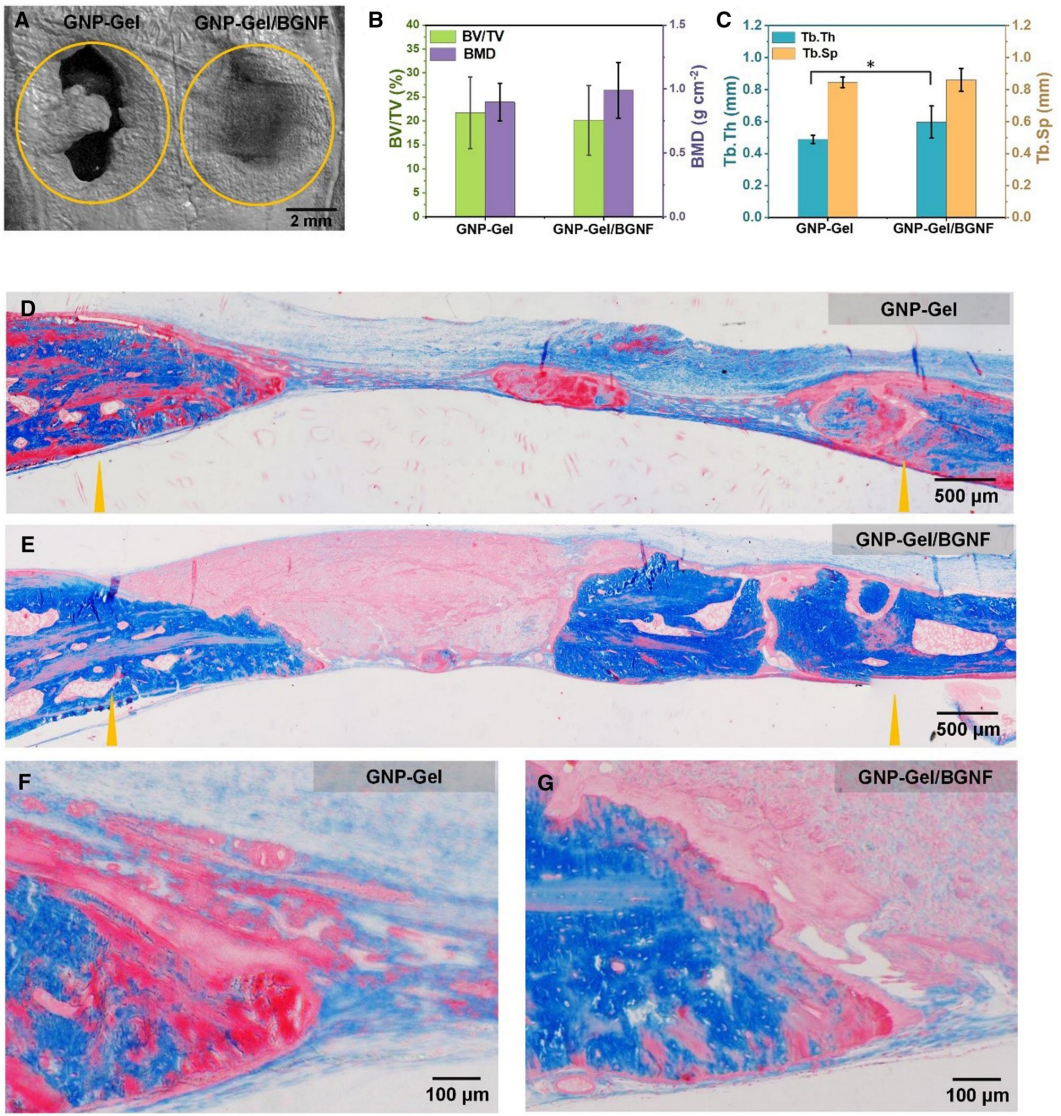
*In vivo* bone regeneration after 12 weeks of repair. (**A**) Micro-CT image of calvaria defects. (**B**) BV/TV and BMD and (**C**) Tb.Th and Tb.Sp of new bone. (**D–G**) Masson staining of new bone. Data are presented as mean ± SD. *P <* *0.05, **P <* *0.01.

The *in vivo* bone regeneration properties of GNP-Gel/BGNF 3D matrixes were evaluated by rat calvaria defect models, and the GNP-Gel 3D matrixes were taken as the control group. The micro-CT images of calvaria defects after 12 weeks of repair are displayed in Figure 5A, and new generated bone in both defects was seen along the defect margin. The quantitative analysis is shown in Figure 5B and C. The average bone volume fractions (BV/TV) of GNP-Gel and GNP-Gel/BGNF 3D matrix groups were 20.12% and 21.71%, respectively. The mean BMD of bone regenerative sections was 0.898 and 0.99 g cm^−3^ for GNP-Gel and GNP-Gel/BGNF 3D matrix groups, respectively. Trabecular thickness (Tb.Th) of GNP-Gel/BGNF 3D matrix group (0.597 mm) was little higher than that of GNP-Gel 3D matrix group (0.489 mm), and the trabecular space (Tb.Sp) was similar to that of GNP-Gel 3D matrix group. Bone regenerative sections were further analyzed by Masson’s staining after 12 weeks of repair (Figure 5D–F). The blue represents the mature bone and the red represents the bone tissue. It can be seen from the Masson’s staining images of GNP-Gel matrix group that new generated bone and bone tissue formed at both the edge and center of the bone defect and the GNP-Gel matrix had degraded. In the GNP-Gel/BGNF 3D matrix group, mature bone generated at the edge of the bone defect and small piece of bone tissue generated at the center of the bone defect, and the GNP-Gel/BGNF 3D matrix was squeezed to the center of the bone defect. Thus, it could be concluded from these data that the BGNFs in the GNP-Gel/BGNF 3D matrix were benefit for the formation of mineralized bone, but the degradation rate was slower than GNP-Gel 3D matrix, which hindered the new bone generation at the center of the bone defects.

## Conclusion

In conclusion, a kind of GNP-Gel/BGNF nanofibrous 3D matrixes were successfully developed for postoperative treatment of osteosarcoma. 80Si BGNFs calcinated at 800°C were chosen as the basic blocks of GNP-Gel/BGNF 3D matrixes for their good flexibility and bioactivity. The GNP-Gel/BGNF 3D matrixes exhibited excellent biocompatibility, and the 3D matrix with 4% genipin showed better mechanical property and photothermal performance, and its photothermal inhibition rate of tumor was 37.7%, and photothermal inhibition rate of *S. aureus* and *B. coli* was 92% and 93%, respectively. The *in vivo* bone repair experiment indicated the GNP-Gel/BGNF 3D matrix was benefit for the formation of mineralized bone, but the degradation rate of GNP-Gel/BGNF 3D matrix should be further optimized. Therefore, implanting of nanofibrous 3D matrix with photothermal therapeutic effect into bone defect after tumor resection is a kind of promising postoperative treatment of osteosarcoma.

## Supplementary Material

rbae088_Supplementary_Data
