# Supplementary material for: Multifunctional 3D matrixes based on flexible bioglass nanofibers for potential application in postoperative therapy of osteosarcoma
Source: Regen Biomater. 2024 Jul 24;11:rbae088. doi: 10.1093/rb/rbae088 (PMC11333569; doi:10.1093/rb/rbae088)
Supplement: rbae088_Supplementary_Data [file rbae088_supplementary_data.docx]

**Supplementary Data**

**Multifunctional 3D Matrixes Based on Flexible Bioglass Nanofibers for Potential Application in** **Postoperative Therapy of Osteosarcoma**

*Lihuan Wang ^a ,b^,* *Liting Yuan ^a^, Yanbing Dong ^a^, Wenli Huang ^a^, Jichang Zhu ^a^, Xuexian Du ^a^, Chenglin Zhang ^a^, Pengbi Liu ^a^,* *Jinpeng Mo ^a^, Bingyan Li ^a^, Zijin Liu ^a^, Xi Yu ^a ,b,^*, and Hui Yu ^a ,b,^**

*^a^* Guangdong-Hong Kong Joint Laboratory for New Textile Materials, School of Textile Science and Engineering, Wuyi University, Jiangmen, 529020, China

*^b^* Guangdong Laboratory of Chemistry and Fine Chemical Industry Jieyang Center, Jieyang, 515200, China.

*Corresponding authors: Dr. Xi Yu (yuxi143@163.com), Prof. Hui Yu (yuhuihui_2000@163.com)


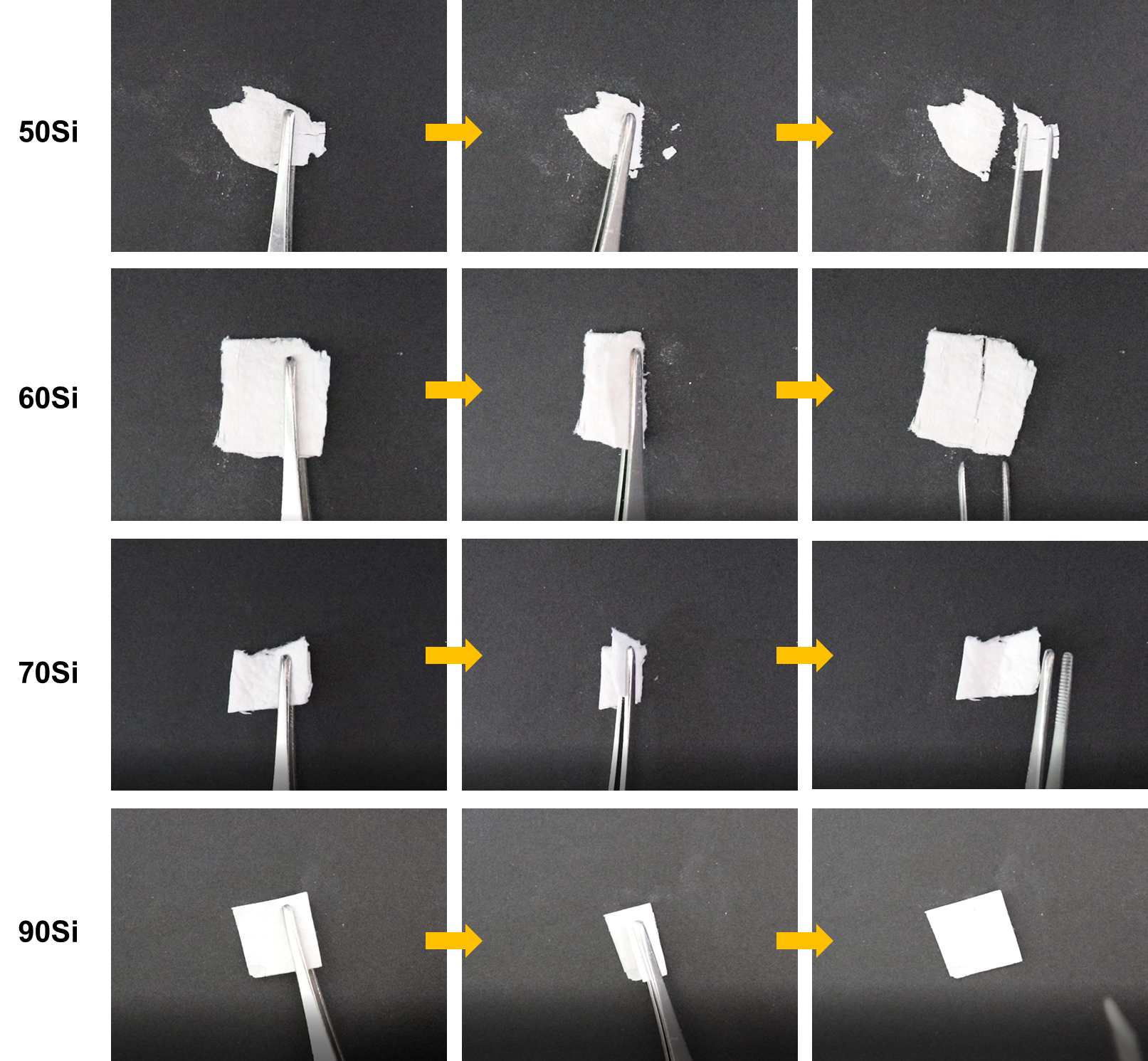


**Figure S1.**  The flexibility display of BGNF membranes.


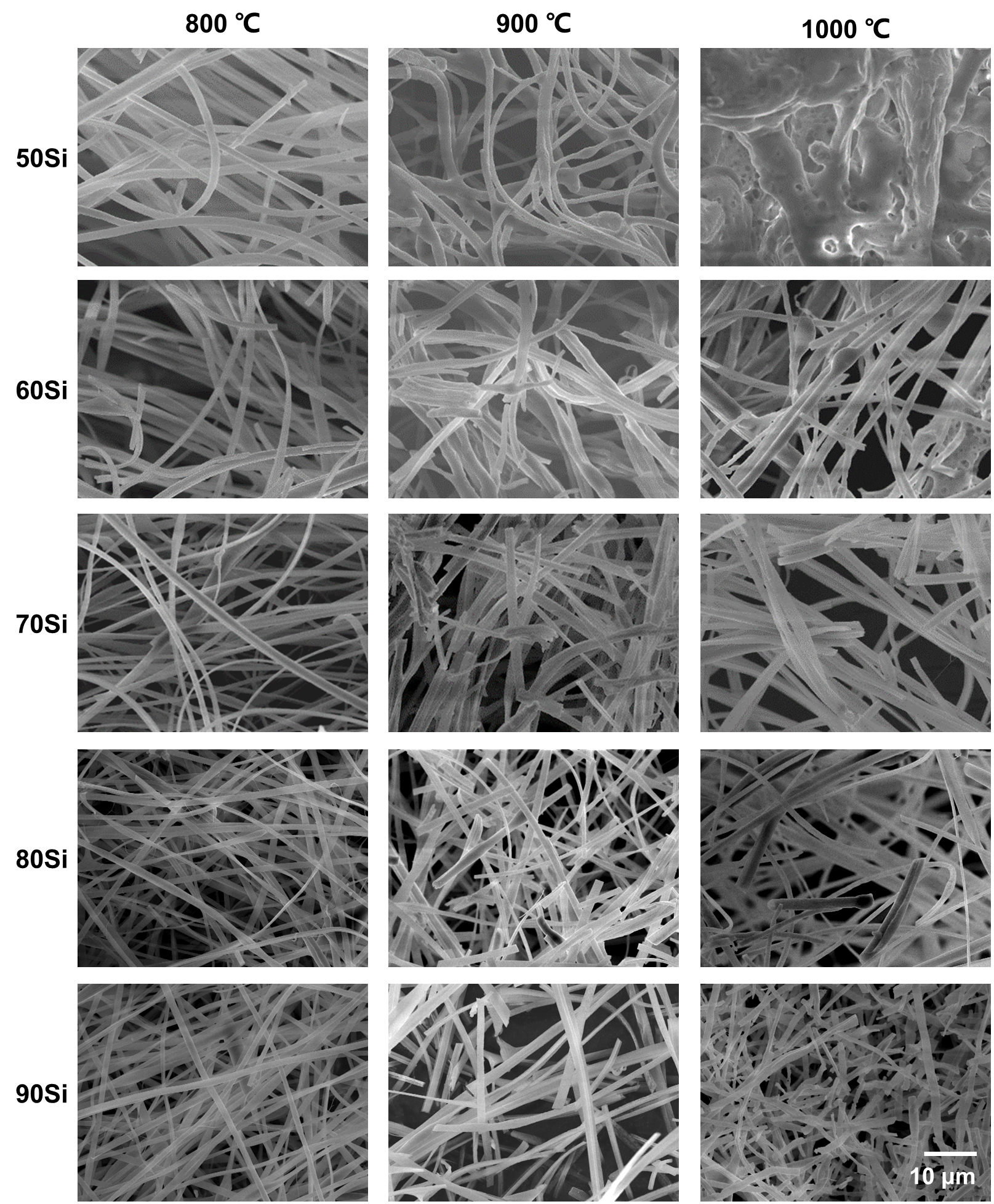


**Figure S2.** The SEM images BGNFs calcinated at different temperatures.


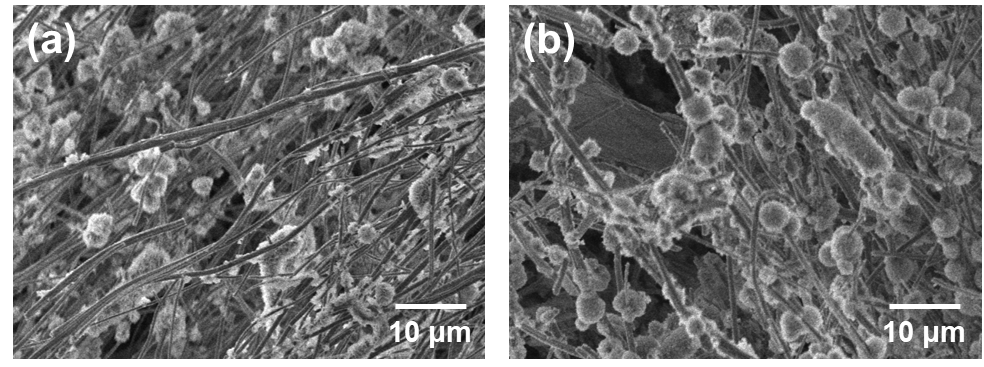


**Figure S3**. SEM images of minerals on 80Si BGNFs calcinated at (a) 900 ^o^C and (b) 1000 ^o^C.


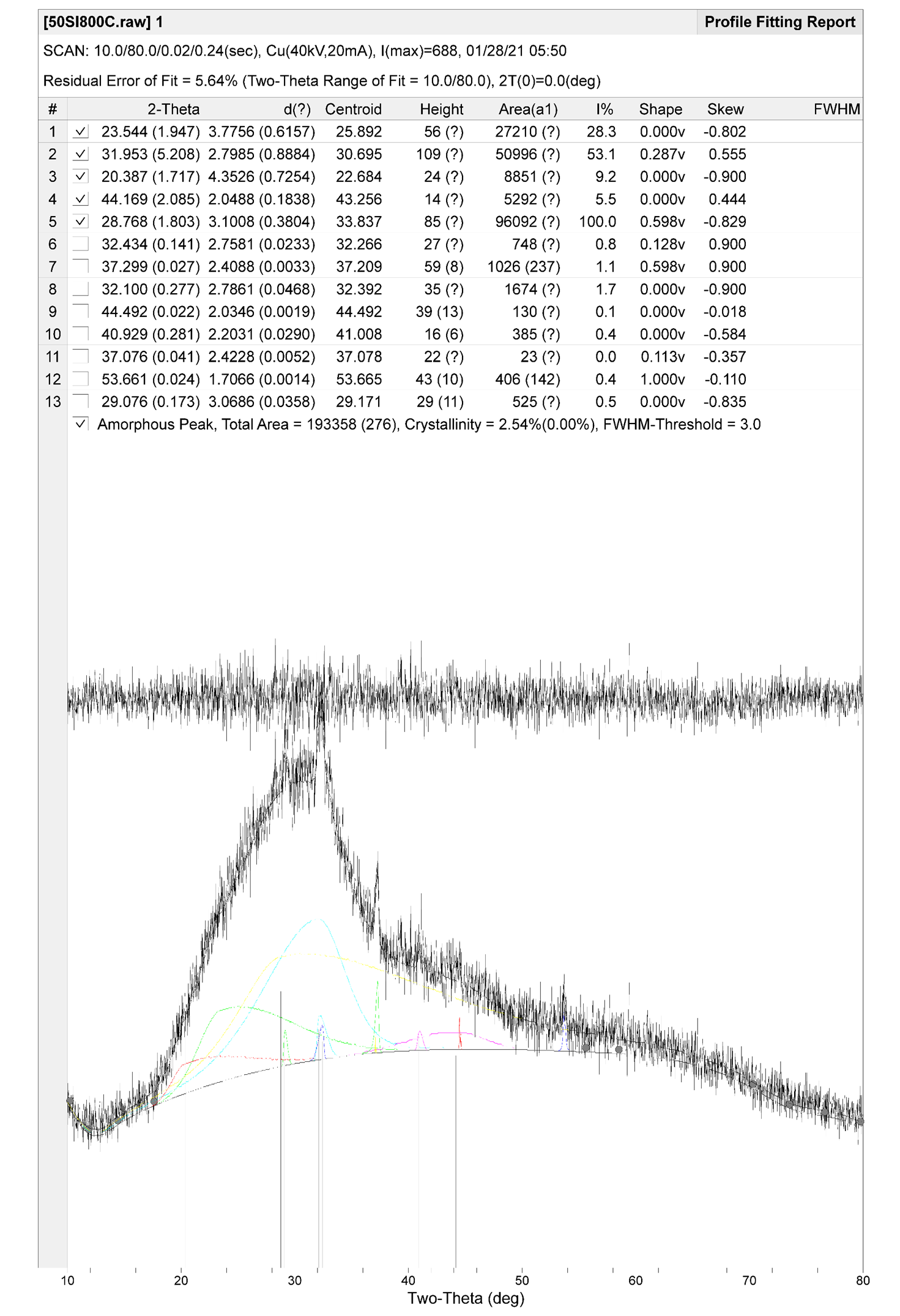


**Figure S4**. XRD profile fitting report of 50Si BGNFs calcinated at 800 ^o^C.


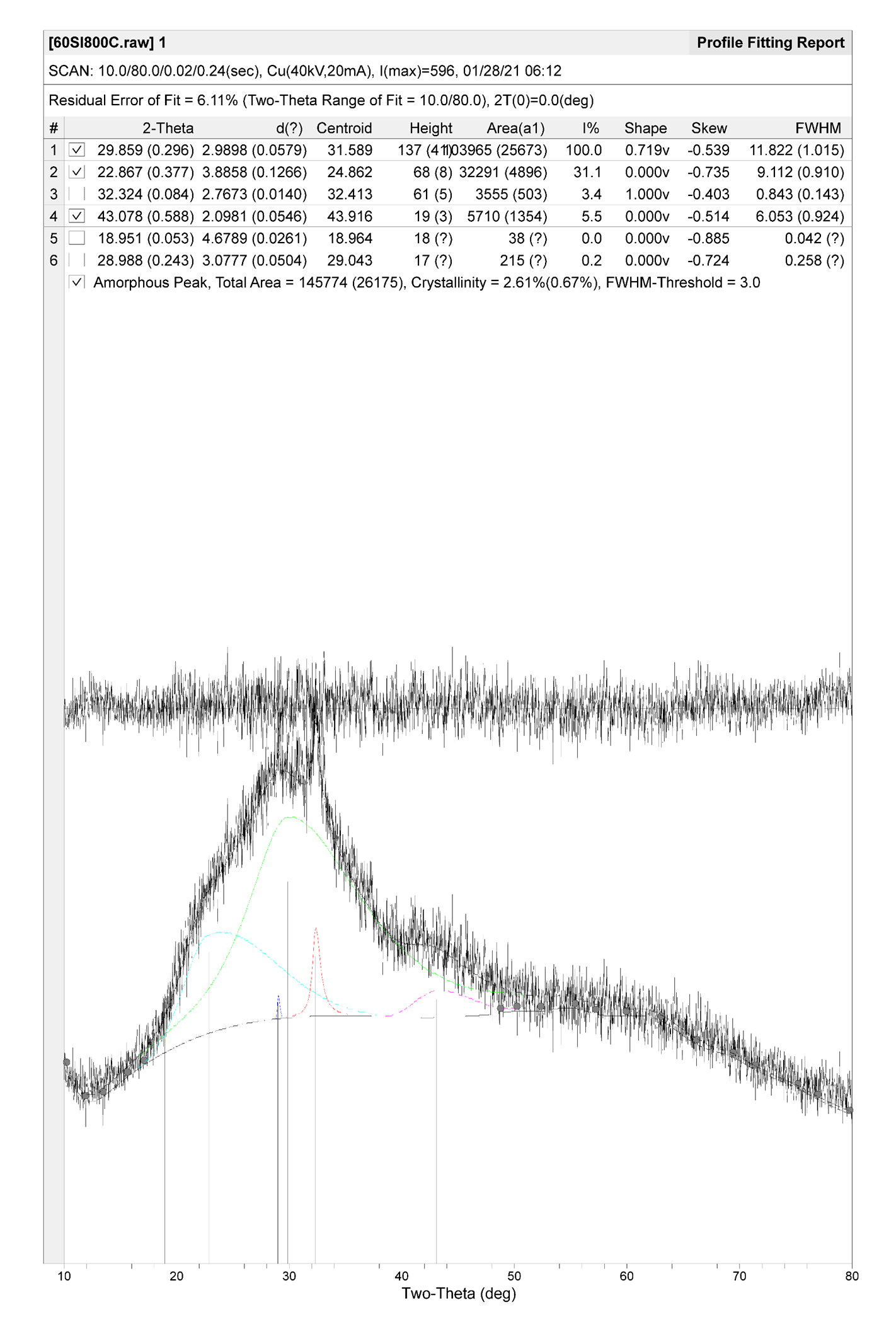


**Figure S5**. XRD profile fitting report of 60Si BGNFs calcinated at 800 ^o^C.


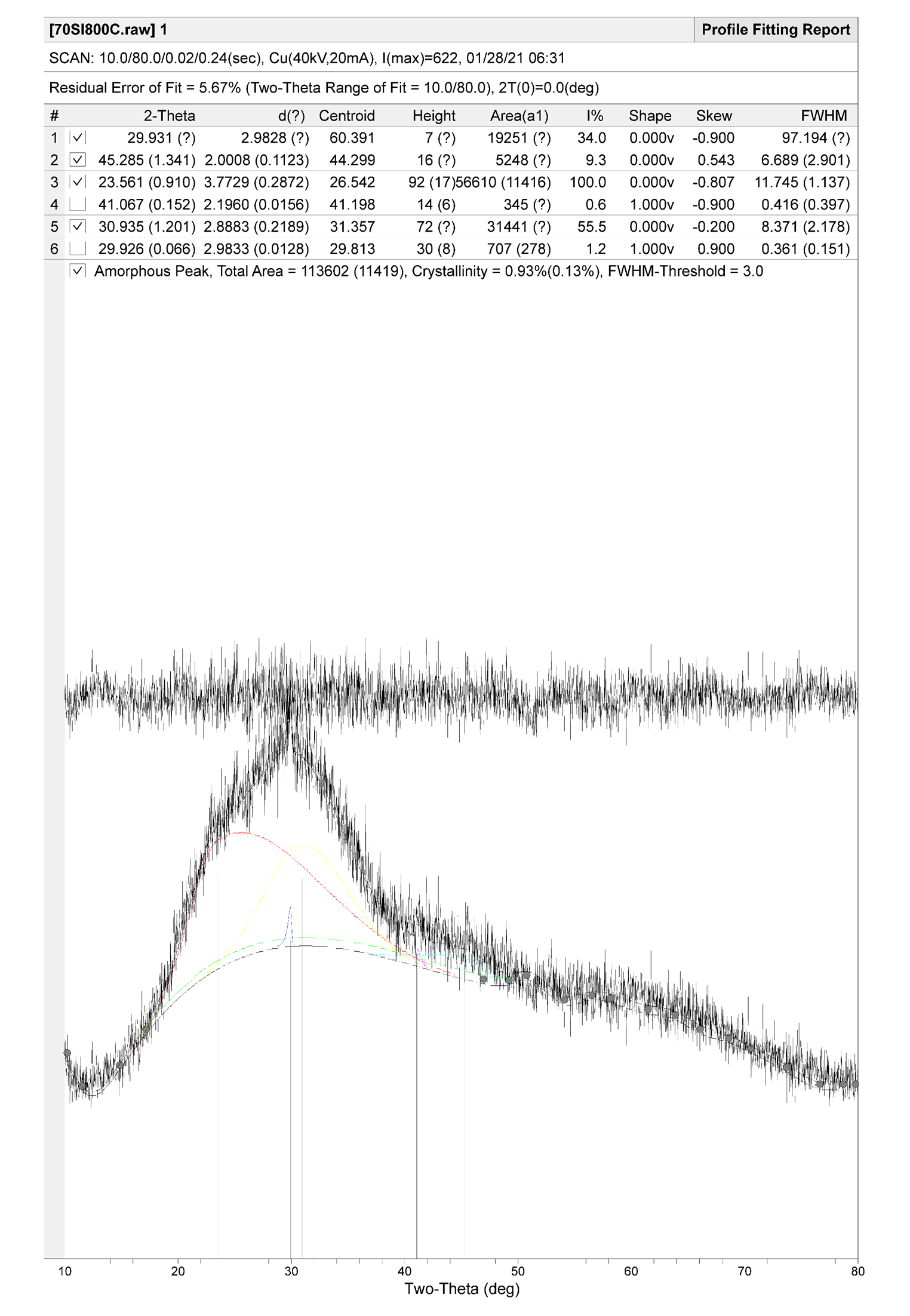


**Figure S6**. XRD profile fitting report of 70Si BGNFs calcinated at 800 ^o^C.


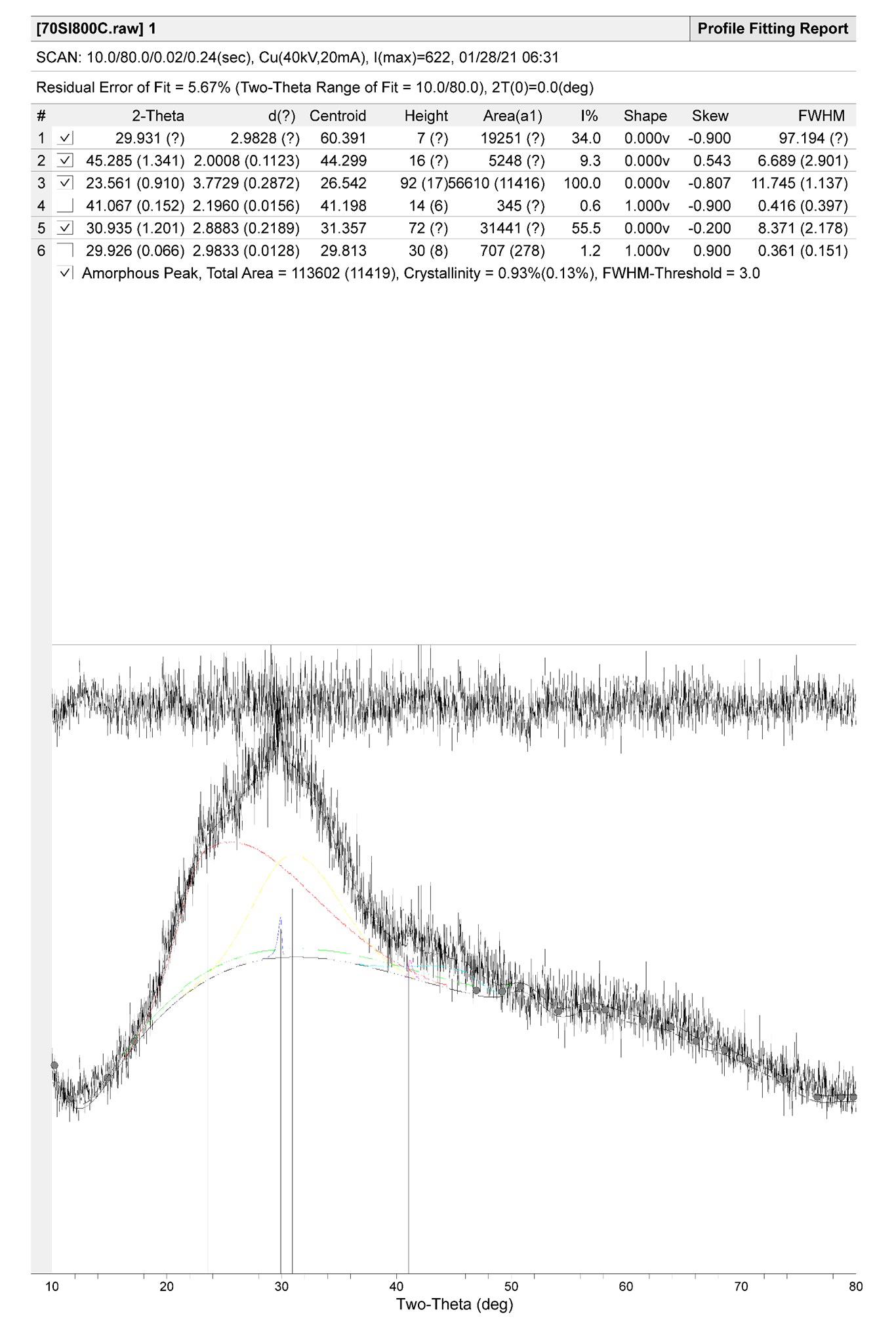


**Figure S7**. XRD profile fitting report of 80Si BGNFs calcinated at 800 ^o^C.


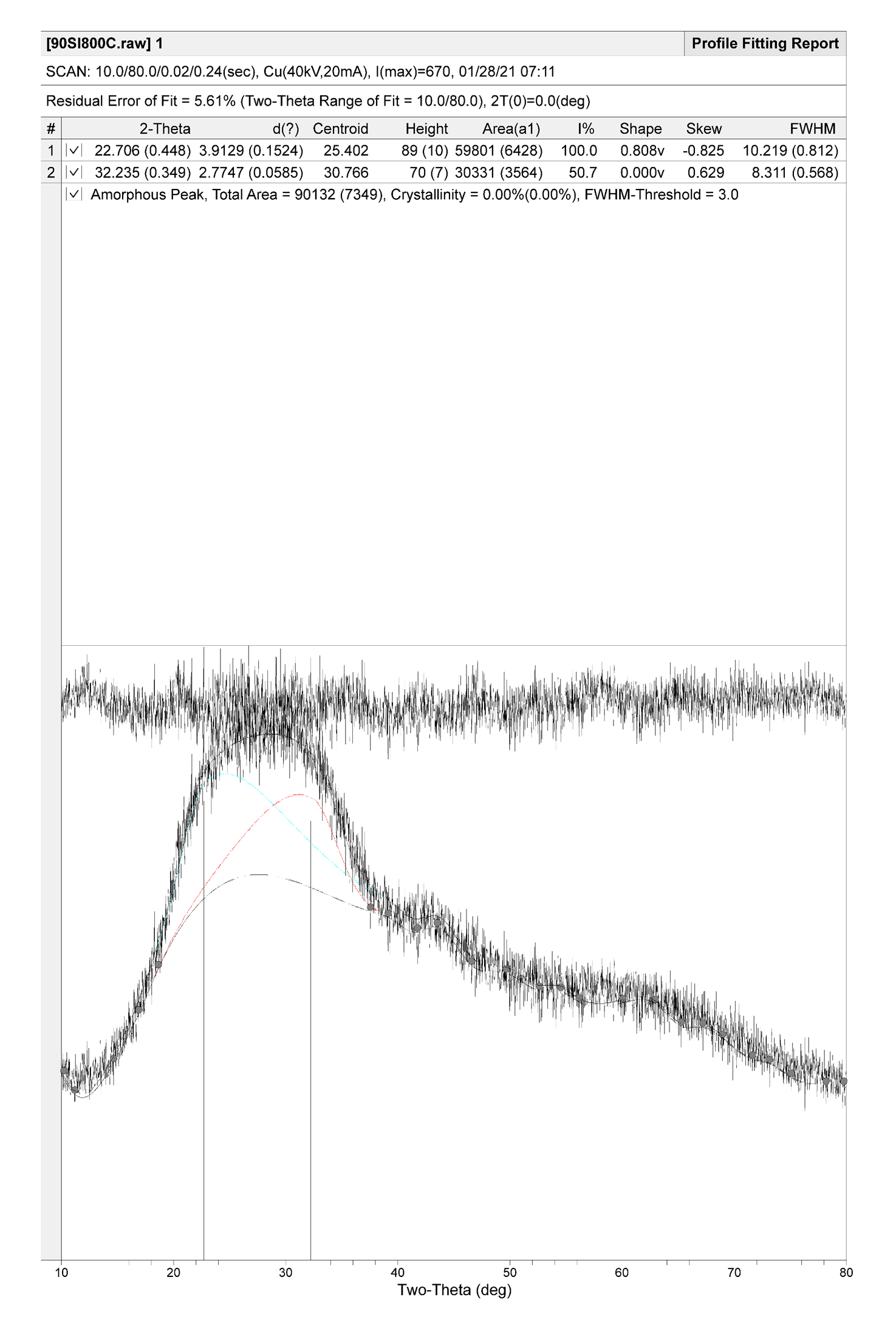


**Figure S8**. XRD profile fitting report of 90Si BGNFs calcinated at 800 ^o^C.


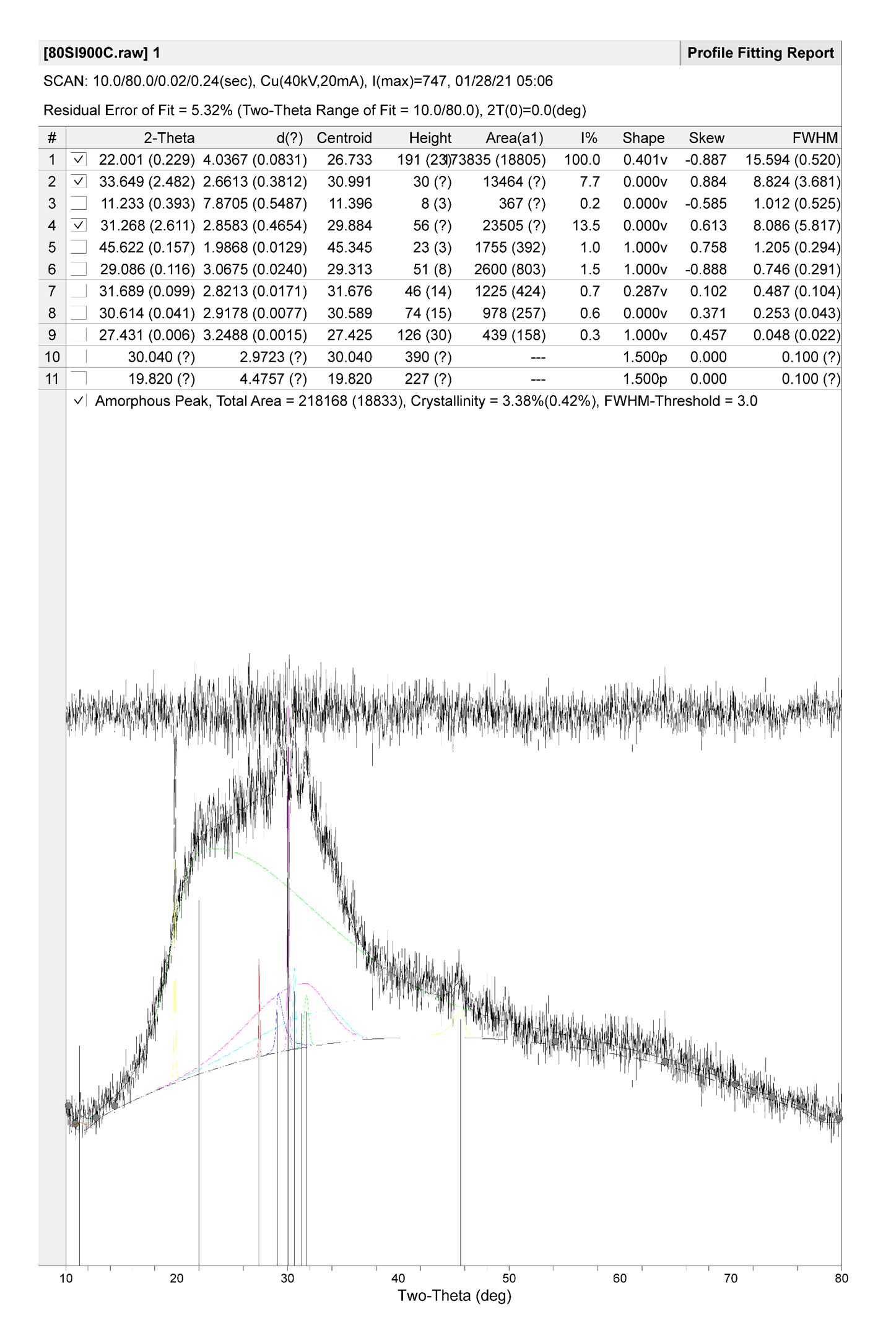


**Figure S9**. XRD profile fitting report of 80Si BGNFs calcinated at 900 ^o^C.


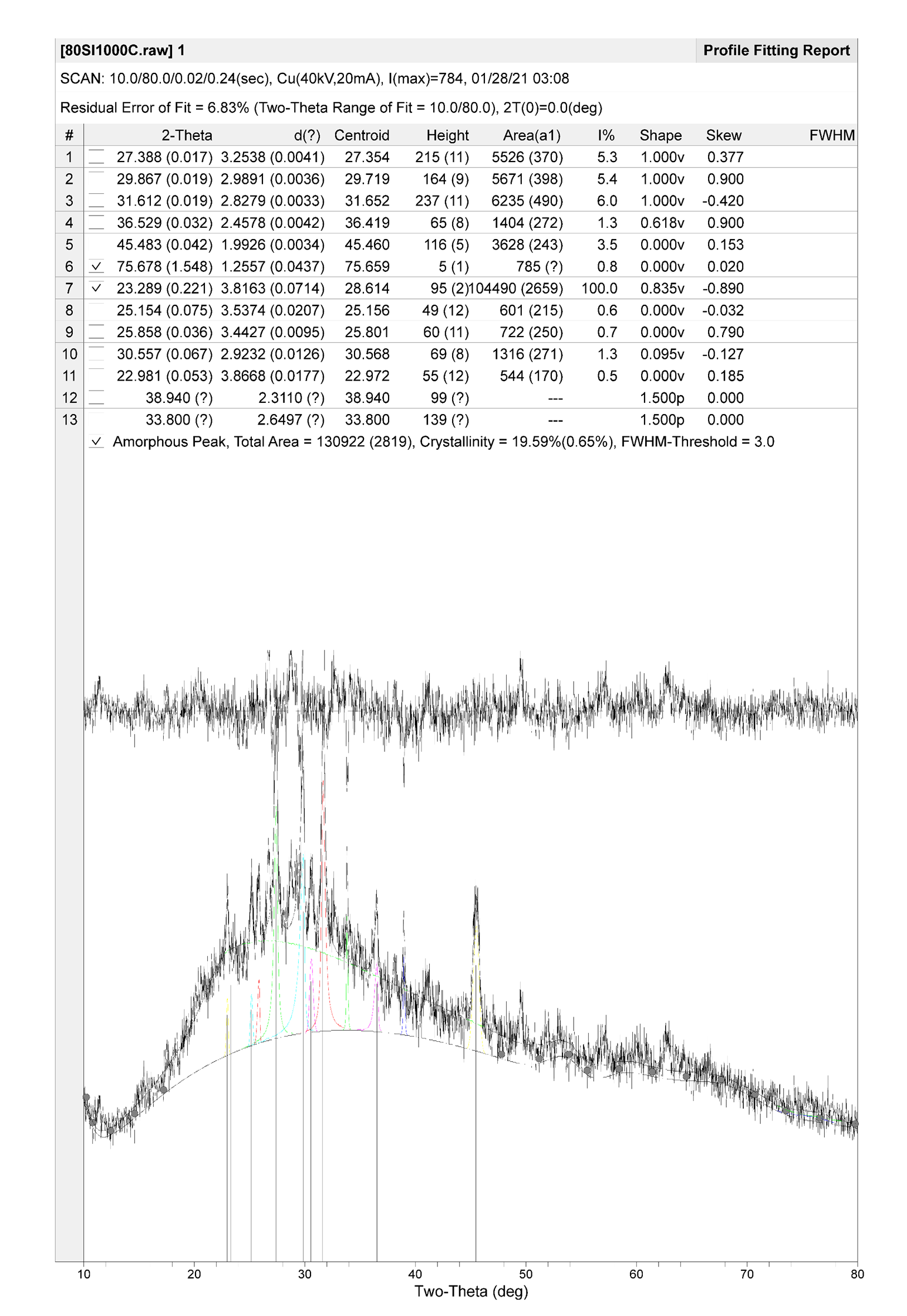


**Figure S10**. XRD profile fitting report of 80Si BGNFs calcinated at 1000 ^o^C.


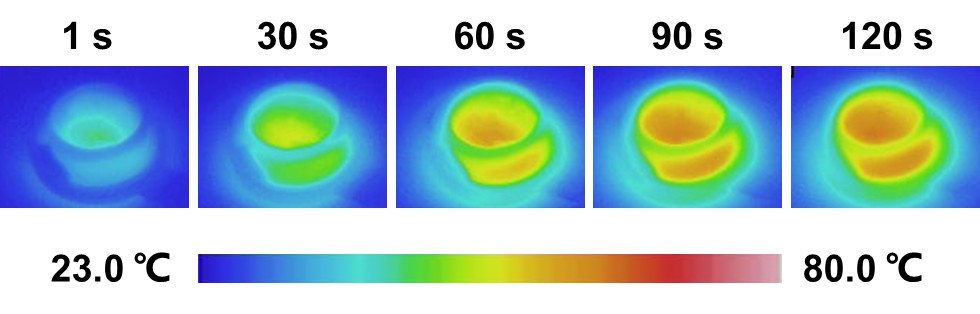


**Figure S11**. Photothermal heating process of GNP-Gel/BGNF 3D matrix with 8% [genipin](https://www.sciencedirect.com/topics/pharmacology-toxicology-and-pharmaceutical-science/genipin) in water.
